# 3-Acetyl­benzoic acid

**DOI:** 10.1107/S1600536810021094

**Published:** 2010-06-05

**Authors:** David E. Fixler, Jacob M. Newman, Roger A. Lalancette, Hugh W. Thompson

**Affiliations:** a Carl A. Olson Memorial Laboratories, Department of Chemistry, Rutgers University, Newark, NJ 07102, USA

## Abstract

In the crystal structure of the title compound, C_9_H_8_O_3_, essentially planar mol­ecules [the carboxyl group makes a dihedral angle of 4.53 (7)° with the plane of the ring, while the acid group forms a dihedral angle of 3.45 (8)° to the ring] aggregate by centrosymmetric hydrogen-bond pairing of ordered carboxyl groups. This yields dimers which have two orientations in a unit cell, creating a herringbone pattern. In addition, two close C—H⋯O inter­molecular contacts exist: one is between a methyl H atom and the ketone of a symmetry-related mol­ecule and the other involves a benzene H atom and the carboxyl group O atom of another mol­ecule. The crystal studied was a non-merohedral twin with twin law [100, 0
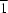
0, 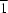
0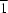] and a domain ratio of 0.8104(14): 0.1896(14).

## Related literature

For a discussion of highly ordered carboxyl bond distances and angles, see: Borthwick (1980 ▶). For the use of the twin law, see: Cooper *et al.* (2002 ▶). For the structure of the *ortho*-isomer, see: Dobson & Gerkin (1996 ▶). For the structure of the *para*-isomer, see: Lalancette *et al.* (2007 ▶).
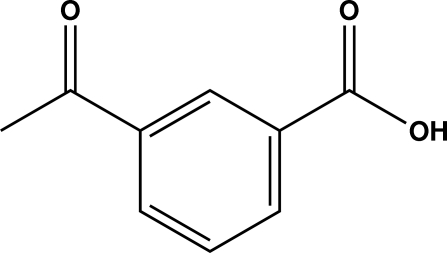


## Experimental

### 

#### Crystal data

C_9_H_8_O_3_
*M* *_r_* = 164.15
Monoclinic, 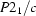

*a* = 3.8202 (1) Å
*b* = 15.6478 (3) Å
*c* = 12.9282 (3) Å
β = 98.508 (1)°
*V* = 764.31 (3) Å^3^
*Z* = 4
Cu *K*α radiation
μ = 0.90 mm^−1^
*T* = 100 K
0.20 × 0.18 × 0.11 mm

#### Data collection

Bruker SMART CCD APEXII area-detector diffractometer
Absorption correction: numerical (*SADABS*; Sheldrick, 2008*a* ▶) *T*_min_ = 0.840, *T*_max_ = 0.907
7201 measured reflections
1386 independent reflections
1351 reflections with *I* > 2σ(*I*)
*R*_int_ = 0.023

#### Refinement

*R*[*F* ^2^ > 2σ(*F* ^2^)] = 0.031
*wR*(*F* ^2^) = 0.085
*S* = 1.06
1386 reflections
115 parameters
H atoms treated by a mixture of independent and constrained refinement
Δρ_max_ = 0.18 e Å^−3^
Δρ_min_ = −0.20 e Å^−3^

### 

Data collection: *APEX2* (Bruker, 2006 ▶); cell refinement: *SAINT* (Bruker, 2005 ▶); data reduction: *SAINT* (Bruker, 2005 ▶); program(s) used to solve structure: *SHELXTL* (Sheldrick, 2008*b* ▶); program(s) used to refine structure: *SHELXTL*; molecular graphics: *SHELXTL*; software used to prepare material for publication: *SHELXTL*.

## Supplementary Material

Crystal structure: contains datablocks I, global. DOI: 10.1107/S1600536810021094/lh5054sup1.cif

Structure factors: contains datablocks I. DOI: 10.1107/S1600536810021094/lh5054Isup2.hkl

Additional supplementary materials:  crystallographic information; 3D view; checkCIF report

## Figures and Tables

**Table 1:** Hydrogen-bond geometry (Å, °)

*D*—H⋯*A*	*D*—H	H⋯*A*	*D*⋯*A*	*D*—H⋯*A*
O3—H3⋯O2^i^	0.931 (19)	1.69 (2)	2.6124 (13)	173.4 (17)
C9—H9*A*⋯O1^ii^	0.98	2.57	3.5283 (17)	167
C4—H4⋯O2^iii^	0.95	2.59	3.3153 (16)	133

Symmetry codes: (i) 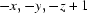; (ii) 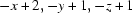; (iii) 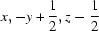.

**Fig. 1.**
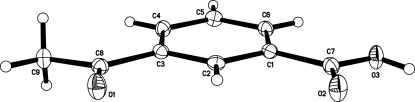
A view of the asymmetric unit of (I) with its numbering scheme. Displacement ellipsoids are drawn at the 40% probability level for non-H atoms.

**Fig. 2.**
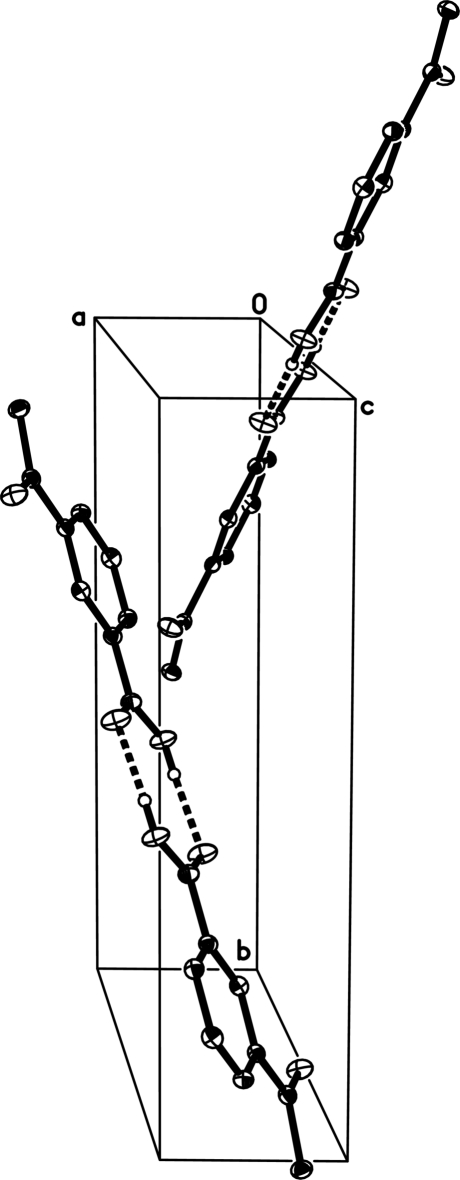
A partial packing diagram showing the herringbone pattern of the two sets of dimers making up this structure. Displacement ellipsoids are drawn at the 40% probability level for non-H atoms. Hydrogen bonds are shown as dashed lines.

## supplementary crystallographic information

### Comment

Since keto carboxylic acids can crystallize in five different hydrogen-bonding modes, these types of compounds always present a challenge to predict *a priori* what the H-bonding mode will be. Three of these modes are either relatively rare or have geometries which preclude some structures. Racemates crystallize with a center of symmetry and generally produce an H-bonded dimer between the acid groups.

Fig. 1 presents a view of the asymmetric unit of the title compound (I) with its numbering scheme. The conjugation in this δ-keto acid requires that both the acid and ketone groups are close to the ring plane; the carboxyl (C3—C8—C9—O1) makes a dihedral angle of 4.53 (7)° with the plane of the ring, while the acid (C1—C7—O2—O3) forms a dihedral angle of 3.45 (8)° to the ring (in the same direction). The molecule adopts a chiral conformation by the flexing of both the methyl group and the hydroxyl group away from the plane of the rest of the atoms in the same direction; this induced chirality is due to the packing of the molecules. This flexing then generates a total dihedral angle between the carboxyl and the acetyl groups of 6.19 (8)°.

Complete or partial averaging of C—O bond lengths and C—C—O angles due to disorder in carboxyl dimers was not found in (I), where these lengths and angles (Table 1) are similar to those in other highly ordered carboxyl situations (Borthwick, 1980).

This meta-acetylbenzoic acid has centrosymmetric H-bonded dimer pairs across two different cell edges in the chosen cell, at (0,0,1/2) & at (0,1/2,0). The parallel planes making up the dimer pair are offset from each other by 0.36 Å. Two sets of these dimers are screw-related and form a herringbone angle of 46.15 (3)° between them in the chosen cell (see Fig 2). Two close C—H···O intermolecular contacts exist: one is between a methyl H atom and the ketone of the adjacent molecule, and the 2nd one is from a phenyl H atom to the carboxyl O atom of another molecule.

Compound (I) crystallizes as a centrosymmetric dimer, just as its isomer 4-acetylbenzoic acid (Lalancette *et al.*, 2007). Unlike both the 3-acetyl and the 4-acetylbenzoic acids, 2-acetylbenzoic acid crystallizes in the phthalide form with a single H-bond betwen the hydroxyl of one molecule and the ketone of the adjacent molecule (Dobson & Gerkin, 1996).

This monoclinic crystal is non-merohedrally twinned; the program ROTAX was used to find the twin law, which was [100, 0-10, -10-1] (Cooper *et al.*, 2002). The final refinement resulted in a ratio of 0.8104 (14): 0.1896 (14) for the two domains, with a final wR2 = 0.085 and R1 = 0.031.

### Experimental

Compound (I) was purchased from Acros Organics, Geel, Belgium. X-ray quality crystals were obtained by evaporation from formic acid at room temperature. The solid-state (KBr) infrared spectrum of (I) features a single broad asymmetric peak at 1686 cm^-1^ for both C=O functions, typical of unstrained carboxyl-paired keto acids. In CHCl_3_ solution, this combined absorption is seen at the same wavenumber.

### Refinement

All H atoms for (I) were found in electron density difference maps. The fractional coordinates of the acid H was allowed to refine and its U_iso_(H) was set at 1.5U_eq_(O). The methyl H atoms were put in ideally staggered positions with C—H distances of 0.98 Å and U_iso_(H) = 1.5U_eq_(C). The phenyl Hs were constrained to ride on their parent C atoms with C—H distances of 0.95 Å and U_iso_(H) = 1.2U_eq_(C).

### Crystal data

**Table:**

C_9_H_8_O_3_	*F*(000) = 344
*M**_r_* = 164.15	*D*_x_ = 1.427 Mg m^−^^3^
Monoclinic, *P*2_1_/*c*	Melting point: 438 K
Hall symbol: -P 2ybc	Cu *K*α radiation, λ = 1.54178 Å
*a* = 3.8202 (1) Å	Cell parameters from 6415 reflections
*b* = 15.6478 (3) Å	θ = 4.5–70.3°
*c* = 12.9282 (3) Å	µ = 0.90 mm^−^^1^
β = 98.508 (1)°	*T* = 100 K
*V* = 764.31 (3) Å^3^	Rod, colourless
*Z* = 4	0.20 × 0.18 × 0.11 mm

### Data collection

**Table:**

Bruker SMART CCD APEXII area-detector diffractometer	1386 independent reflections
Radiation source: fine-focus sealed tube	1351 reflections with *I* > 2σ(*I*)
graphite	*R*_int_ = 0.023
φ and ω scans	θ_max_ = 71.0°, θ_min_ = 2.8°
Absorption correction: numerical (*SADABS*; Sheldrick, 2008a)	*h* = −4→4
*T*_min_ = 0.840, *T*_max_ = 0.907	*k* = −18→18
7201 measured reflections	*l* = −15→15

### Refinement

**Table:**

Refinement on *F*^2^	Secondary atom site location: difference Fourier map
Least-squares matrix: full	Hydrogen site location: inferred from neighbouring sites
*R*[*F*^2^ > 2σ(*F*^2^)] = 0.031	H atoms treated by a mixture of independent and constrained refinement
*wR*(*F*^2^) = 0.085	*w* = 1/[σ^2^(*F*_o_^2^) + (0.0497*P*)^2^ + 0.2254*P*] where *P* = (*F*_o_^2^ + 2*F*_c_^2^)/3
*S* = 1.06	(Δ/σ)_max_ < 0.001
1386 reflections	Δρ_max_ = 0.18 e Å^−^^3^
115 parameters	Δρ_min_ = −0.20 e Å^−^^3^
0 restraints	Extinction correction: *SHELXTL* (Sheldrick, 2008b), Fc^*^=kFc[1+0.001xFc^2^λ^3^/sin(2θ)]^-1/4^
Primary atom site location: structure-invariant direct methods	Extinction coefficient: 0.0029 (9)

### Special details

**Table:**

Experimental. 'crystal mounted on a Cryoloop using Paratone-N'
Geometry. All esds (except the esd in the dihedral angle between two l.s. planes) are estimated using the full covariance matrix. The cell esds are taken into account individually in the estimation of esds in distances, angles and torsion angles; correlations between esds in cell parameters are only used when they are defined by crystal symmetry. An approximate (isotropic) treatment of cell esds is used for estimating esds involving l.s. planes.
Refinement. Refinement of *F*^2^ against ALL reflections. The weighted *R*-factor wR and goodness of fit *S* are based on *F*^2^, conventional *R*-factors *R* are based on *F*, with *F* set to zero for negative *F*^2^. The threshold expression of *F*^2^ > σ(*F*^2^) is used only for calculating *R*-factors(gt) etc. and is not relevant to the choice of reflections for refinement. *R*-factors based on *F*^2^ are statistically about twice as large as those based on *F*, and *R*- factors based on ALL data will be even larger.

### Fractional atomic coordinates and isotropic or equivalent isotropic displacement parameters (Å^2^)

**Table:**

	*x*	*y*	*z*	*U*_iso_*/*U*_eq_	
O1	0.7469 (3)	0.37754 (6)	0.50800 (7)	0.0258 (3)	
C1	0.1930 (4)	0.17156 (8)	0.35852 (10)	0.0167 (3)	
O2	0.2316 (3)	0.09082 (6)	0.51488 (7)	0.0272 (3)	
C2	0.3766 (3)	0.23971 (8)	0.41031 (10)	0.0166 (3)	
H2	0.4582	0.2359	0.4832	0.020*	
O3	−0.0709 (3)	0.03570 (6)	0.36893 (7)	0.0249 (3)	
H3	−0.127 (5)	−0.0066 (12)	0.4144 (14)	0.037*	
C3	0.4409 (3)	0.31325 (8)	0.35589 (10)	0.0161 (3)	
C4	0.3245 (4)	0.31730 (8)	0.24841 (10)	0.0187 (3)	
H4	0.3691	0.3671	0.2104	0.022*	
C5	0.1437 (4)	0.24901 (9)	0.19657 (10)	0.0193 (3)	
H5	0.0667	0.2524	0.1234	0.023*	
C6	0.0753 (4)	0.17637 (8)	0.25067 (10)	0.0176 (3)	
H6	−0.0505	0.1300	0.2152	0.021*	
C7	0.1179 (4)	0.09528 (8)	0.41951 (11)	0.0187 (3)	
C8	0.6344 (4)	0.38585 (8)	0.41580 (11)	0.0178 (3)	
C9	0.6809 (4)	0.46813 (8)	0.35915 (10)	0.0215 (3)	
H9A	0.8271	0.5075	0.4062	0.032*	
H9B	0.4487	0.4941	0.3364	0.032*	
H9C	0.7972	0.4564	0.2980	0.032*	

### Atomic displacement parameters (Å^2^)

**Table:**

	*U*^11^	*U*^22^	*U*^33^	*U*^12^	*U*^13^	*U*^23^
O1	0.0343 (6)	0.0221 (5)	0.0188 (5)	−0.0065 (5)	−0.0033 (5)	0.0005 (4)
C1	0.0180 (6)	0.0156 (7)	0.0169 (7)	0.0015 (5)	0.0044 (6)	−0.0002 (5)
O2	0.0430 (7)	0.0222 (5)	0.0149 (5)	−0.0086 (5)	−0.0009 (5)	0.0019 (4)
C2	0.0175 (6)	0.0192 (6)	0.0131 (6)	0.0018 (5)	0.0026 (5)	−0.0007 (5)
O3	0.0356 (6)	0.0180 (5)	0.0199 (5)	−0.0088 (5)	0.0001 (5)	−0.0002 (4)
C3	0.0151 (6)	0.0159 (6)	0.0174 (6)	0.0019 (5)	0.0026 (5)	−0.0004 (5)
C4	0.0198 (7)	0.0168 (6)	0.0195 (7)	0.0005 (5)	0.0029 (6)	0.0029 (5)
C5	0.0210 (7)	0.0228 (7)	0.0134 (6)	0.0014 (6)	0.0005 (6)	−0.0003 (5)
C6	0.0177 (7)	0.0176 (6)	0.0172 (7)	−0.0011 (5)	0.0013 (6)	−0.0033 (5)
C7	0.0218 (7)	0.0164 (6)	0.0180 (7)	−0.0007 (5)	0.0031 (6)	−0.0021 (5)
C8	0.0166 (6)	0.0178 (7)	0.0190 (7)	0.0015 (5)	0.0026 (6)	−0.0011 (5)
C9	0.0230 (7)	0.0166 (7)	0.0241 (7)	−0.0033 (6)	0.0012 (6)	0.0007 (5)

### Geometric parameters (Å, °)

**Table:**

O1—C8	1.2128 (17)	C3—C8	1.5057 (18)
C1—C2	1.3922 (19)	C4—C5	1.3897 (19)
C1—C6	1.4022 (18)	C4—H4	0.9500
C1—C7	1.4818 (18)	C5—C6	1.3797 (19)
O2—C7	1.2471 (17)	C5—H5	0.9500
C2—C3	1.3896 (19)	C6—H6	0.9500
C2—H2	0.9500	C8—C9	1.5047 (18)
O3—C7	1.2952 (17)	C9—H9A	0.9800
O3—H3	0.931 (19)	C9—H9B	0.9800
C3—C4	1.3963 (19)	C9—H9C	0.9800
			
C2—C1—C6	120.11 (12)	C5—C6—C1	119.43 (12)
C2—C1—C7	118.98 (11)	C5—C6—H6	120.3
C6—C1—C7	120.89 (12)	C1—C6—H6	120.3
C3—C2—C1	120.34 (12)	O2—C7—O3	123.04 (12)
C3—C2—H2	119.8	O2—C7—C1	120.31 (12)
C1—C2—H2	119.8	O3—C7—C1	116.64 (12)
C7—O3—H3	110.9 (12)	O1—C8—C9	121.32 (12)
C2—C3—C4	119.16 (12)	O1—C8—C3	120.02 (12)
C2—C3—C8	118.33 (11)	C9—C8—C3	118.66 (11)
C4—C3—C8	122.51 (12)	C8—C9—H9A	109.5
C5—C4—C3	120.50 (12)	C8—C9—H9B	109.5
C5—C4—H4	119.8	H9A—C9—H9B	109.5
C3—C4—H4	119.8	C8—C9—H9C	109.5
C6—C5—C4	120.46 (12)	H9A—C9—H9C	109.5
C6—C5—H5	119.8	H9B—C9—H9C	109.5
C4—C5—H5	119.8		
			
C6—C1—C2—C3	0.7 (2)	C7—C1—C6—C5	178.73 (12)
C7—C1—C2—C3	−177.89 (12)	C2—C1—C7—O2	−3.2 (2)
C1—C2—C3—C4	−1.1 (2)	C6—C1—C7—O2	178.21 (13)
C1—C2—C3—C8	178.83 (12)	C2—C1—C7—O3	176.16 (12)
C2—C3—C4—C5	0.6 (2)	C6—C1—C7—O3	−2.4 (2)
C8—C3—C4—C5	−179.34 (13)	C2—C3—C8—O1	4.06 (19)
C3—C4—C5—C6	0.3 (2)	C4—C3—C8—O1	−176.04 (14)
C4—C5—C6—C1	−0.7 (2)	C2—C3—C8—C9	−175.43 (12)
C2—C1—C6—C5	0.1 (2)	C4—C3—C8—C9	4.47 (19)

### Hydrogen-bond geometry (Å, °)

**Table:**

*D*—H···*A*	*D*—H	H···*A*	*D*···*A*	*D*—H···*A*
O3—H3···O2^i^	0.931 (19)	1.69 (2)	2.6124 (13)	173.4 (17)
C9—H9A···O1^ii^	0.98	2.57	3.5283 (17)	167
C4—H4···O2^iii^	0.95	2.59	3.3153 (16)	133

Symmetry codes: (i) −*x*, −*y*, −*z*+1; (ii) −*x*+2, −*y*+1, −*z*+1; (iii) *x*, −*y*+1/2, *z*−1/2.
